# Incidence of Air Leak Syndrome in Pediatric Patients With SARS-COV-2 Pneumonia and Respiratory Failure: A Single-Center Retrospective Study

**DOI:** 10.7759/cureus.43329

**Published:** 2023-08-11

**Authors:** Juan Cardenas, Jose M Cardenas, Matthew Garber, Jose Irazuzta

**Affiliations:** 1 Pediatric Medicine, University of Florida College of Medicine – Jacksonville, Jacksonville, USA; 2 Pediatric Critical Care, University of Florida College of Medicine, Gainesville, USA; 3 Hospital Medicine, University of Florida College of Medicine – Jacksonville, Jacksonville, USA; 4 Pediatric Critical Care, University of Florida College of Medicine – Jacksonville, Jacksonville, USA

**Keywords:** extracorporeal membrane oxygenation support, respiratory failure, covid 19, sars-cov-2, air leak syndrome

## Abstract

Air leak syndrome (ALS) is defined as the extrusion of air from an aerated compartment into an unaerated compartment with associated symptoms of respiratory distress. This syndrome can occur as a consequence of trauma, iatrogenic causes, or spontaneously. Retrospective investigations conducted in the adult population have demonstrated an elevated risk of spontaneous ALS development in patients with coronavirus disease 2019 (COVID-19) pneumonia, along with its correlation with mortality. However, no studies have yet explored this phenomenon within the pediatric population. In light of this knowledge gap, we conducted a retrospective chart review comprising 128 pediatric patients ranging in age from one month to 18 years. The primary objective was to assess the incidence of ALS in two distinct groups: patients diagnosed with COVID-19 pneumonia and those with non-COVID-19 viral pneumonia. The groups were compared using Fisher's exact test for sex, the presence of ALS, the requirement of extracorporeal membrane oxygenation (ECMO), and death. The modified Wald method was used to calculate the 95% confidence interval for the mortality rate in patients with COVID-19 pneumonia in the presence of ALS. Our findings revealed a higher prevalence of ALS in patients with COVID-19 pneumonia compared to the non-COVID-19 viral pneumonia group, with a statistically significant P-value of 0.02 and an odds ratio (OR) of 6.72. In terms of mortality rates, there was a statistically significant difference between the two groups (P = 0.025, OR = 1.083). In addition, in patients with ALS in the presence of COVID-19 pneumonia, the mortality rate was 37.5%. However, the requirement of ECMO was not statistically significant (P = 0.16, OR = 1.04). These results suggest that patients with COVID-19 pneumonia have an increased mortality rate and a heightened risk of developing ALS compared to individuals with other viral pneumonias. Furthermore, the presence of ALS was associated with a high mortality rate in COVID-19 pneumonia patients. However, it is crucial to note that obtaining a larger patient sample and involving multiple institutions would be necessary to obtain more consistent and robust data.

## Introduction

Severe acute respiratory syndrome coronavirus-2 (SARS-CoV-2) is a novel strain of coronavirus, which causes COVID-19. It was discovered in December 2019 and led to a worldwide pandemic after an outbreak of respiratory infections in a seafood market in Wuhan, China [1]. Disease severity is variable, but most cases in children have been asymptomatic. Children with symptomatic disease usually present with fever, cough, rhinorrhea, sore throat, dyspnea, headache, malaise, and gastrointestinal symptoms such as vomiting, diarrhea, and abdominal pain. More recently, children have presented with symptoms of laryngotracheobronchitis. Ten percent of children present with more severe lower respiratory tract symptoms requiring hospital admission, and 0.5-2% require admission to the intensive care unit [2].

SARS-CoV-2 targets the angiotensin-converting enzyme 2 (ACE-2) receptors present in multiple organs including the vascular endothelium, myocardium, proximal tubules of the kidney, and intestines, and mostly affects type II pneumocytes [3]. The effect of SARS-CoV-2 on ACE-2 receptors disrupts the protective function of the renin-angiotensin pathway in the lung provoking endothelial dysfunction that leads to vasoconstriction, increased dead space, and arterial hypoxemia. In addition, endothelial dysfunction provokes thrombotic macro- and microangiopathy preceded by the activation of the coagulation and complement system [4]. These pathobiological effects could explain the high elastance phenotype with decreased compliance and increased lung weight that presents later in the disease [5]. These processes may result in lung damage and alveolar friability, which may lead to the formation of cysts and bullae that increase the risk of having an air leak syndrome (ALS) without the presence of barotrauma [4].

ALS is defined as the extrusion of air from an aerated compartment into an unaerated compartment with associated symptoms of respiratory distress. The incidence of ALS in patients with COVID-19 pneumonia is not completely known [5]. However, retrospective studies done in adults have shown that the incidence is around 1% [6-8]. Other adult multicenter observational studies have demonstrated that the incidence of ALS increases up to 12.8% in critically ill patients requiring invasive mechanical ventilation [9]. Mortality in adults has been related to the presence of ALS in observational studies but may be associated with other factors such as age and comorbidities [9].

The incidence of ALS in COVID-19 pneumonia has not been studied in detail in the pediatric population. There are no studies other than case reports describing ALS in pediatric patients with COVID-19 pneumonia [10,11]. We observed several pediatric cases of unexpected ALS early in the pandemic and wondered if there was a propensity for this complication. Our primary aim was to compare the incidence of ALS in critically ill pediatric patients with COVID-19 pneumonia to critically ill pediatric patients with non-COVID-19 viral pneumonia. Our secondary aim was to compare clinical outcomes among these two groups, the mortality rate in children with COVID-19 pneumonia and ALS, as well as age and sex differences between patients with and without ALS.

## Materials and methods

We conducted an observational, retrospective study at Wolfson Children’s Hospital in Jacksonville, Florida, USA, an American College of Surgeons level one trauma center containing an open-heart program, burn program, extracorporeal membrane oxygenation (ECMO), and continuous renal replacement therapy. Its 26-bed quaternary-level pediatric intensive care unit (PICU) has 1491 annual admissions, with a mortality of 2.7% (n=41) and a standardized mortality ratio (PRISM III) between 0.71 and 1.11. This study was exempted by the Institutional Review Board of Baptist Health.

We searched the electronic medical records from January 1st, 2016, to December 31st, 2021, to identify patients aged one month to 18 years with a diagnosis of respiratory failure secondary to viral pneumonia. This was confirmed at admission with an upper respiratory panel by polymerase chain reaction (PCR) obtained via a nasal swab. Viruses that were identified include SARS-CoV-2, human metapneumovirus, respiratory syncytial virus, adenovirus, rhinovirus, enterovirus, influenza type A and B, parainfluenza type 1, 2 and 3, and non-COVID-19 coronavirus. All identified patients with COVID-19 pneumonia from the beginning of the pandemic until December 31st, 2021, were included. Although no formal power calculation was performed, we retrospectively searched electronic medical records in reverse chronological order until we obtained at least one viral pneumonia case for every COVID-19 case because not enough controls were available during the pandemic.

Patients were divided into a COVID-19 pneumonia group and a control group affected with pneumonia due to other non-COVID-19 viral etiologies. No patient had COVID-19 and another virus simultaneously. We compared age, gender, the presence of air leak confirmed by chest X-ray, the provision of ECMO, and death between the COVID-19 pneumonia group and the non-COVID-19 viral pneumonia group, as well as age and gender between patients with and without ALS using the Fisher’s exact test. We computed odds ratios and 95% confidence intervals for these outcomes. The median age difference between groups and between patients with and without ALS was compared using the Mann-Whitney U test. We calculated the mortality rate in patients with ALS from COVID-19 and its 95% confidence interval using the modified Wald method. All statistical tests were two-sided with a P-value <0.05 considered statistically significant. Data were analyzed using Statistical Package for the Social Sciences (SPSS) version 29 (IBM Corp, Armonk, NY).

## Results

One hundred and twenty-eight patients, who were admitted to the PICU between January 1st, 2016, and December 31, 2021, and who met the inclusion criteria, were identified. Of those, 52 patients were diagnosed with COVID-19 pneumonia and 76 were diagnosed with viral pneumonia in the absence of SARS-CoV-2. Sex, age, and outcomes for each group are presented in Table 1. For the COVID-19 pneumonia group, 8 (16%) experienced ALS, of which two patients required ECMO support due to severe acute respiratory distress syndrome and subsequently died. A third patient died without receiving ECMO support because the patient's parents refused escalation of care. In the non-COVID-19 viral pneumonia group, two patients (2.6%) experienced air leaks, and none died or required ECMO support.

**Table 1:** Patient Demographics and Outcomes IQR: interquartile range; CI: confidence interval; ECMO: extracorporeal membrane oxygenation. *Mann-Whitney U test median difference.

	All patients (n=128)	SARS-CoV-2 (n=52) n (%)	Non-SARS-CoV-2 (n=76) n (%)		P-value [odds ratio (95% CI)]
Males %	65 (50.7%)	28 (53%)		37 (48%)	0.59 [1.23 (0.61-2.50)]
Median age in months (IQR)	48 (147)	168 (141)		24 (45)	<0.001[ 130 (144-84)]*
Air leak %	10 (7.8%)	8 (15%)		2 (2.6%)	0.02 [6.72 (1.37-33.11)]
ECMO %	2 (1.5%)	2 (3.8%)		0 (0%)	0.16 [1.04 (0.99-1.10)]
Death %	3 (2.3%)	3 (5.7%)		0% (0%)	0.025 [1.083 (CI 1.00-1.17)]

ALS was more common in patients with COVID-19 pneumonia in comparison with other non-COVID-19 viral pneumonias with a P-value of 0.02 and odds ratio of 6.72 (CI 1.37-33.11). Similarly, death was more common in patients with COVID-19 pneumonia with a P-value of 0.03 and odds ratio of 1.083 (CI 1.00-1.17). ECMO support only occurred in patients with COVID-19 pneumonia; however, the difference was not significant (P-value 0.16). Mortality occurred in three of the eight patients with air leaks in the presence of COVID-19 pneumonia, for a mortality rate of 37.5% and 95% confidence interval of 13%-70%. ALS did not differ by age (P=0.11) or sex (P=1.0). 

## Discussion

Our data show that COVID-19 pneumonia may have a predisposition to the development of ALS at a rate higher than other viral pneumonias. We also found a higher mortality rate in critically ill pediatric patients with COVID-19 pneumonia versus patients with other viral pneumonias. This difference was statistically significant despite our small sample size. In the adult population, ALS is more frequently seen in males between day 9 and day 19 of hospitalization, which corresponds with the time frame of a high pulmonary elastance phenotype. This is characterized by poor lung compliance and higher lung weight, which may predispose the patient to self-inflicted lung injury (P-SILI) and the development of ALS [9,12,13]. In our study, no statistical significance was found for male predominance.

A statistically significant relationship was observed in relation to age. This finding could potentially be attributed to the prevalence of asymptomatic or mildly symptomatic SARS-CoV-2 infections in children, which generally do not require admission to the intensive care unit. Conversely, viral pneumonia tends to primarily affect children below the age of five years [14,15].

In our investigation, it was difficult to utilize timing as a clinical metric to establish causality between high elastance phenotype and the development of ALS. Given that COVID-19 pneumonia is less severe in the pediatric population in comparison with the adult population, our patients may have presented to the hospital later in the disease and, therefore, the ALS may have originated prior to admission [2].

In a multicenter retrospective study done in adults, the presence of ALS was correlated with a mortality rate as high as 36%. While our sample size was small, we observed a similar mortality rate for patients with ALS due to COVID-19 pneumonia [16]. In the event that ALS development is linked to significant mortality, all possible actions should be taken into account to prevent ALS.

We are unaware of any studies in children showing a higher need for ECMO in patients with air leaks in the presence of COVID-19 pneumonia. Two of our 52 patients with COVID-19 pneumonia and ALS required ECMO compared with none in the non-SARS-CoV-2 group. While not statistically different, this may represent a type II error given our small sample size. The potential increased need for ECMO in these patients may be related to the severity of the disease, comorbidities, or intrinsic pathobiology of the air leak.

In the adult literature, ALS has been described in patients not exposed to baro- or volu-trauma, as well as an increased risk in patients on non-invasive positive ventilation [7,17,18]. The pathobiology of SARS-CoV-2-induced ALS is not completely understood; however, the loss of the protective effect of ACE-2 inhibitors and an increase in the tidal volume in the context of hypoxia could lead to P-SILI, followed by ALS. While this would imply an impairment of the Herring Breuer reflex which would limit lung hyperinflation to prevent self-injury, this remains a matter of speculation [4,13,19].

In addition to being a retrospective review, our study has some limitations. These patients come from a single institution, so results may not be generalizable to other institutions based on mechanical practice patterns. Our sample size is small and we may be underpowered to detect true differences between the groups. We did not collect data on tidal volumes and intra-esophageal pressures. Other than age and sex, we did not risk stratify by comorbid conditions or other patient factors.

## Conclusions

Patients with COVID-19 pneumonia appear to be at a higher risk of developing ALS and have higher mortality in comparison with other viral pneumonias. It is important to consider that the mortality rate is significantly higher in patients with air leaks in the presence of COVID-19 pneumonia. Therefore, the heightened risk should be considered in these cases, and strategies to prevent ALS should be considered due to the associated rise in mortality. A better understanding of ALS in patients with COVID-19 pneumonia and its repercussions will allow the development of targeted treatment strategies. We are currently performing a multi-institutional study with a larger patient population to further evaluate the incidence of ALS in patients with COVID-19 pneumonia and further explore the impact of SARS-CoV-2 on patient outcomes such as the need for ECMO and mortality.
